# Reservoirs of Non-*baumannii Acinetobacter* Species

**DOI:** 10.3389/fmicb.2016.00049

**Published:** 2016-02-01

**Authors:** Ahmad Al Atrouni, Marie-Laure Joly-Guillou, Monzer Hamze, Marie Kempf

**Affiliations:** ^1^Laboratoire Microbiologie Santé et Environnement, Centre AZM pour la Recherche en Biotechnologie et ses Applications, Ecole Doctorale des Sciences et de Technologie, Université LibanaiseTripoli, Liban; ^2^ATOMycA, Inserm Atip-Avenir Team, CRCNA, Inserm U892, 6299 Centre National de la Recherche Scientifique, University of AngersAngers, Lebanon; ^3^Laboratoire de Bactériologie, Institut de Biologie en Santé – Centre Hospitalier UniversitaireAngers, France; ^4^Faculté de Santé Publique, Université LibanaiseTripoli, Lebanon

**Keywords:** *Acinetobacter* spp., non-*baumannii*, extra-hospital reservoirs, environment, humans, animals, food, novel species

## Abstract

*Acinetobacter* spp. are ubiquitous gram negative and non-fermenting coccobacilli that have the ability to occupy several ecological niches including environment, animals and human. Among the different species, *Acinetobacter baumannii* has evolved as global pathogen causing wide range of infection. Since the implementation of molecular techniques, the habitat and the role of non-*baumannii Acinetobacter* in human infection have been elucidated. In addition, several new species have been described. In the present review, we summarize the recent data about the natural reservoir of non-*baumannii Acinetobacter* including the novel species that have been described for the first time from environmental sources and reported during the last years.

## Introduction

Implementation of molecular techniques in research laboratories has greatly improved the identification of *Acinetobacter* species. Among these techniques, 16S-rRNA, RNA polymerase subunit B (*rpoB*), and DNA gyrase subunit B (*gyrB*) gene sequencing, as well as DNA-DNA hybridization and whole genome sequencing provide good informative data for *Acinetobacter* taxonomic studies (Rafei et al., 2014; Jung and Park, 2015). Based on these methods, novel species have been reported and the genus now contains 51 species with valid published names (http://apps.szu.cz/anemec/Classification.pdf. (Accessed October, 2015).

*Acinetobacter* species are ubiquitous in nature and can be found in different environmental sources such as hydrocarbon contaminated areas, activated sludge, sewage, dump sites, but also on vegetables, animals, and humans (Doughari et al., 2011). The ability to dominate in so many ecological niches led thus some authors to consider these bacteria as microbial weeds (Cray et al., 2013).

Among the different species, *Acinetobacter baumannii* is the leading one. It has emerged in recent decades as a clinically relevant pathogen causing a wide range of nosocomial infections, community-acquired infections or war and natural disaster-related infections (Peleg et al., 2008). Nevertheless, the role of non-*baumannii Acinetobacter* in human infections is increasingly reported thanks to technological advances such as molecular biology that allow correct identification of the bacteria at the species level. Thus, for example, several cases concerning multidrug resistant *Acinetobacter pittii* and *Acinetobacter nosocomialis* strains that caused infections in health-care facilities have been reported around the world (Karah et al., 2011; Kouyama et al., 2012; Yang et al., 2012; Schleicher et al., 2013; Fitzpatrick et al., 2015). *Acinetobacter calcoaceticus* which is mainly an environmental species has been described in several cases of pneumonia and bacteraemia (Mostachio et al., 2012; Li et al., 2015a), and nosocomial infections due to species like *Acinetobacter lwoffii, Acinetobacter junii*, or *Acinetobacter johnsonii* were also reported (Lee et al., 2007; Karah et al., 2011).

Because of its important role in human infections, *A. baumannii* has been the most studied bacterium of the *Acinetobacter* genus. In contrast, little is known on other *Acinetobacter* species. The present review aims to summarize the recent data of non-*baumannii Acinetobacter* with a focus on the natural reservoir, and including the novel species that have been described for the first time from environmental sources and reported during the last years by using molecular techniques (Table 1).

**Table 1:** **Natural habitat of non-*baumannii Acinetobacter* species**.

***Acinetobacter* species**	**Origin of isolation**	**Country of isolation**	**Identification method**	**References**
*A. albensis*	Water, soil	Czech Republic	Phenotypic, 16S-RNA, *gyrB, rpoB, gltA, pyrG, recA*, Maldi-TOF	Krizova et al., 2015a
*A. anitratus*	Animal	France	Phenotypic, 16S-rRNA	La Scola et al., 2001
*A. antiviralis*	Plant roots	Korea	% G+C, fatty acid analysis, 16S-RNA, DNA-DNA hybridization	Lee et al., 2009
*A. apis*	Animal	Korea	DNA-DNA hybridization, 16S rRNA gene and *rpoB* sequence analysis, % G+C, and fatty acid analysis	Kim et al., 2014
*A. baylyi*	Activated sludge	Australia	16S-rRNA DNA-DNA hybridization	Carr et al., 2003
*A. beijerinckii*	Animal	Lebanon	*rpoB*	Rafei et al., 2015
*A. bereziniae*	Sewage	Denmark	16S-rRNA	Geiger et al., 2009
	Life environment surface	Korea	16S-rRNA *rpoB*	Choi et al., 2012
	Vegetables	Hong Kong UK	ARDRA	Berlau et al., 1999a; Houang et al., 2001
	Meat	Lebanon	*rpoB*	Rafei et al., 2015
	Human skin	Germany Hong Kong	Phenotypic, ARDRA, SDS-PAGE, ribotyping, DNA-DNA hybridization, RAPD	Seifert et al., 1997; Chu et al., 1999
	Animal	Lebanon	*rpoB*	Rafei et al., 2015
*A. bohemicus*	Soil	Czech Republic	*rpoB, gyrB*, 16S-rRNA	Krizova et al., 2014
	Water	Czech Republic	*rpoB, gyrB*, 16S-rRNA	Krizova et al., 2014
*A. boissieri*	Floral nectar	Spain	Phenotypic, G+C, fatty acids, 16S-rRNA, *rpoB*, DNA-DNA hybridization	Álvarez-Pérez et al., 2013
*A. bouvetii*	Activated sludge	Australia	16S-rRNA DNA-DNA hybridization	Carr et al., 2003
*A. brisouii*	Wetland (Peat)	Korea	Phenotypic, G+C, fatty acids, 16S-rRNA, DNA-DNA hybridization	Anandham et al., 2010
*A. calcoaceticus*	Sewage, water	Denmark, Croitia	16S-rRNA	Geiger et al., 2009; Maravić et al., 2015
	Soil	Hong Kong Korea Lebanon China	ARDRA 16S-rRNA *rpoB*	Houang et al., 2001; Choi et al., 2012; Rafei et al., 2015; Wang and Sun, 2015
	Vegetables	Lebanon UK	*rpoB* ARDRA	Berlau et al., 1999a; Rafei et al., 2015; Al Atrouni et al., 2015
	Animal	Lebanon	*rpoB*	Rafei et al., 2015
	Human skin	Hong Kong India	Phenotypic, ARDRA, RAPD	Chu et al., 1999; Patil and Chopade, 2001
*A. gandensis*	Water	Croitia	–	Maravić et al., 2015
	Animal	–	Phenotypic, DNA-DNA hybridization, 16S rRNA *rpoB*, % G+C, fatty acid, MALDI-TOF MS	Smet et al., 2014
*A. gerneri*	Activated sludge	Australia	16S-rRNA DNA-DNA hybridization	Carr et al., 2003
	Animal	Lebanon	*rpoB*	Rafei et al., 2015
*A. grimontii*,	Activated sludge	Australia	16S-rRNA DNA-DNA hybridization	Carr et al., 2003
*A. guangdongensis*	lead-zinc ore mine site	China	Phenotypic, G+C, fatty acids, 16S-rRNA, *gyrB, rpoB*, DNA-DNA hybridization	Feng et al., 2014b
*A. guillouiae*	Water	Denmark	16S-rRNA	Geiger et al., 2009
	Vegetables	UK	ARDRA	Berlau et al., 1999a
	Human skin	Hong Kong UK, Netherland	Phenotypic, ARDRA, RAPD, AFLP	Chu et al., 1999; Dijkshoorn et al., 2005
*A. haemolyticus*	Water	Croitia	–	Maravić et al., 2015
	Human skin	India	Phenotypic	Patil and Chopade, 2001
*A. harbinensis*	Water	China	Phenotypic, G+C, fatty acids, 16S-rRNA, *gyrB, rpoB*, DNA-DNA hybridization	Li et al., 2014b
*A. indicus*	Dump site	India	Phenotypic, G+C, fatty acids, 16S-rRNA, *rpoB*, DNA-DNA hybridization	Malhotra et al., 2012
*A. johnsonii*	Activated sludge	Germany	Pcr fingerprinting	Wiedmann-al-Ahmad et al., 1994
	Sewage, water, sea food	Denmark, Croitia, China	16S-rRNA	Geiger et al., 2009; Zong and Zhang, 2013; Maravić et al., 2015
	Animal	Lebanon	*rpoB*	Rafei et al., 2015
	Human skin	Germany Hong Kong UK, Netherland	Phenotypic, ARDRA, SDS-PAGE, ribotyping, DNA-DNA hybridization, RAPD, AFLP	Seifert et al., 1997; Chu et al., 1999; Dijkshoorn et al., 2005
*A. junii*	Activated sludge	Germany	Pcr fingerprinting	Wiedmann-al-Ahmad et al., 1994
	Sewage, water	Denmark, Croitia	16S-rRNA	Geiger et al., 2009; Maravić et al., 2015
	Animal	Lebanon	*rpoB*	Rafei et al., 2015
	Soil	China	ARDRA 16S-rRNA *rpoB*	Wang and Sun, 2015
	Human skin	Germany Hong Kong India UK, Netherland	Phenotypic, ARDRA, SDS-PAGE, ribotyping, DNA-DNA hybridization, RAPD, AFLP	Seifert et al., 1997; Chu et al., 1999; Patil and Chopade, 2001; Dijkshoorn et al., 2005
*A. koukii*	Soil, beet field, sediment	Korea, Germany, Netherland, Malaysia, Thailand	Phenotypic, G+C, fatty acids, 16S-rRNA, *gyrB, rpoB*, DNA-DNA hybridization	Choi et al., 2013
*A. kyonggiensis*	Sewage	Korea	Phenotypic, G+C, fatty acids, 16S-rRNA, DNA-DNA hybridization	Lee and Lee, 2010
*A. lwoffii*	Activated sludge	Germany	PCR fingerprinting	Wiedmann-al-Ahmad et al., 1994
	Sewage, water, sea food	Denmark	16S-rRNA	Geiger et al., 2009
	Life environment surface	Korea	16S-rRNA *rpoB*	Choi et al., 2012
	Animal	Lebanon, Croitia	*rpoB*, 16S-RNA	Rafei et al., 2015; Sun et al., 2015
	Vegetables	UK	ARDRA	Berlau et al., 1999a
	Human skin	Germany UK Hong Kong India	Phenotypic, ARDRA, SDS-PAGE, ribotyping, DNA-DNA hybridization, RAPD	Seifert et al., 1997; Berlau et al., 1999b; Chu et al., 1999; Patil and Chopade, 2001
*A. marinus*	Water	Korea	G+C, 16S-RNA, DNA-DNA hybridization	Yoon et al., 2007
*A. nectaris*	Floral nectar	Spain	Phenotypic, G+C, fatty acids, 16S-rRNA, *rpoB*, DNA-DNA hybridization	Álvarez-Pérez et al., 2013
*A. nosocomialis*	Sewage	Denmark	16S-rRNA	Geiger et al., 2009
	Life environment surface	Korea	16S-rRNA *rpoB*	Choi et al., 2012
	Vegetables	UK	ARDRA	Berlau et al., 1999a
	Human skin	Hong Kong	ARDRA, RAPD	Chu et al., 1999
*A. oleivorans*	Rice paddy	Korea	% G+C, fatty acid analysis, 16S-RNA, DNA-DNA hybridization	Kang et al., 2011
*A. pakistanensis*	Wastewater	Pakistan	Phenotypic, fatty acids, 16S-rRNA, *gyrB, rpoB, atpD*, DNA-DNA hybridization	Abbas et al., 2014
*A. parvus*	Soil	Korea	16S-rRNA *rpoB*	Choi et al., 2012
	Life environment surface	Korea	16S-rRNA *rpoB*	Choi et al., 2012
*A. pittii*	Sewage	Denmark	16S-rRNA	Geiger et al., 2009
	Soil	Hong Kong, Lebanon	ARDRA *rpoB*	Houang et al., 2001; Rafei et al., 2015
	Vegetables	Hong Kong Lebanon UK	ARDRA *rpoB*	Berlau et al., 1999a; Houang et al., 2001; Rafei et al., 2015
	Life environment surface	Korea	16S-rRNA *rpoB*	Choi et al., 2012
	Water	Lebanon	*rpoB*	Rafei et al., 2015
	Cheese, Meat	Lebanon	*rpoB*	Rafei et al., 2015
	Animal	Lebanon	*rpoB*	Rafei et al., 2015
	Human skin	Germany Hong Kong India	Phenotypic, ARDRA, SDS-PAGE, ribotyping, DNA-DNA hybridization, RAPD	Seifert et al., 1997; Chu et al., 1999; Patil and Chopade, 2001
*A. populi*	Populus bark	China	Phenotypic,16S-RNA, *gyrB, rpoB*, DNA-DNA hybridization	Li et al., 2015b
*A. puyangensis*	Populus bark	China	Phenotypic, G+C, fatty acids, 16S-rRNA, *gyrB, rpoB*, DNA-DNA hybridization	Li et al., 2013
*A. qingfengensis*	Populus bark	China	Phenotypic, G+C, fatty acids, 16S-rRNA, *gyrB, rpoB*, DNA-DNA hybridization	Li et al., 2014a
*A. radioresistens*	Soil, cotton, water	Australia, Croitia		Dortet et al., 2006; Maravić et al., 2015
	Life environment surface	Korea	16S-rRNA *rpoB*	Choi et al., 2012
	Animal	Lebanon	*rpoB*	Rafei et al., 2015; Sunantaraporn et al., 2015
	Human skin	Germany UK Hong Kong	Phenotypic, ARDRA, SDS-PAGE, ribotyping, DNA-DNA hybridization, RAPD	Seifert et al., 1997; Berlau et al., 1999b; Chu et al., 1999
*A. refrigeratoris*	Life environment surface	China	16S-rRNA, *rpoB* DNA-DNA hybridization	Feng et al., 2014a
*A. rudis*	Wastewter, raw milk	Portugal, Israel	Phenotypic, G+C, fatty acids, 16S-rRNA, *gyrB, rpoB*, DNA-DNA hybridization	Vaz-Moreira et al., 2011
*A. seifertii/genomspecies close 13 TU*	Life environment surface	Korea	16S-RNA, *rpoB*	Choi et al., 2012
	Human skin	Hong Kong	ARDRA, RAPD	Chu et al., 1999
*A. seohaensis*	Water	Korea	G+C, 16S-RNA, DNA-DNA hybridization	Yoon et al., 2007
*A. shindleri*	Life environment surface	Korea	16S-rRNA *rpoB*	Choi et al., 2012
	Animal	Lebanon	*rpoB*	Rafei et al., 2015; Sunantaraporn et al., 2015
*A. soli*	Soil	Korea	Phenotypic, fatty acids, G+C content, 16S-rRNA gyrB, DNA-DNA hybridization	Kim et al., 2008
	Life environment surface	Korea	16S-rRNA *rpoB*	Choi et al., 2012
	Vegetables	Lebanon	*rpoB*	Rafei et al., 2015
*A. tandoii*	Activated sludge plant	Australia	16S-rRNA DNA-DNA hybridization	Carr et al., 2003
	Soil	Korea	16S-rRNA *rpoB*	Choi et al., 2012
	Life environment surface	Korea	16S-rRNA *rpoB*	Choi et al., 2012
*A. tjernbergiae*	Activated sludge	Australia	16S-rRNA DNA-DNA hybridization	Carr et al., 2003
*A. towneri*	Activated sludge	Australia	16S-rRNA DNA-DNA hybridization	Carr et al., 2003
*A. variabilis /(genomspecies 15TU)*	Sewage, water, sea food	Denmark	16S-rRNA	Geiger et al., 2009
	Life environment surface	Korea	rpoB	Choi et al., 2012
	Human skin	Hong Kong	ARDRA, RAPD	Chu et al., 1999
	Animal	France	Phenotypic, *gyrA, gyrB, rpoB*	Poirel et al., 2012
	Animal	–	Phenotypic, *rpoB*, gyrB, Maldi-Tof, whole genome analysis	Nishimura et al., 1988
*A. venetianus*	Water Oil vegetables	Israel, Italy, Denmark, Hong Kong, japan	Phenotypic, DNA-DNA hybridization, AFLP, *rpoB*, ARDRA, tDNA PCR	Vaneechoutte et al., 2009
*Acinetobacter* spp.	Water	China Malaysia, Thailand Vietnam	16S-rRNA	Fuhs and Chen, 1975; Huys et al., 2007; Krizova et al., 2015b; Xiong et al., 2015
	Soil	France-Kuwait	16S-rRNA	Bordenave et al., 2007; Obuekwe et al., 2009
	Meat	Hong Kong	ARDRA	Houang et al., 2001
	Fish, shrimps	Hong Kong	ARDRA	Houang et al., 2001; Huys et al., 2007
	Sediment	China Malaysia, Thailand Vietnam	16S-rRNA	Huys et al., 2007; Xiong et al., 2015
	Plants nectar	Israel, Spain	Pyrosequencing, 16S-rRNA	Fridman et al., 2012; Álvarez-Pérez and Herrera, 2013
	Milk	United states Kenya Korea	Phenotypic	Jayarao and Wang, 1999; Ndegwa et al., 2001; Nam et al., 2009; Gurung et al., 2013
	Animal	Angola	16S-rRNA	Guardabassi et al., 1999
	Human skin	Germany Hong Kong India UK, Netherland	Phenotypic, ARDRA, SDS-PAGE, ribotyping, DNA-DNA hybridization, RAPD, AFLP	Seifert et al., 1997; Chu et al., 1999; Patil and Chopade, 2001; Dijkshoorn et al., 2005
*genomspecies 14 BJ*	Sewage	Denmark	16S-rRNA	Geiger et al., 2009
	Human skin	Hong Kong	ARDRA, RAPD	Chu et al., 1999
*A. genospecies 15 BJ*	Human skin	UK Hong Kong	Phenotypic, ADRA, RAPD	Berlau et al., 1999b; Chu et al., 1999
*genomspecies 16*	Sewage	Denmark	16S-rRNA	Geiger et al., 2009
	Vegetables	Hong Kong	ARDRA	Houang et al., 2001
	Human skin	Hong Kong	ARDRA, RAPD	Chu et al., 1999
*A. genospecies 17*	Human skin	Hong Kong	ARDRA, RAPD	Chu et al., 1999
*A. genospecies 13 BJ, 14 TU*	Human skin	Hong Kong	ARDRA, RAPD	Chu et al., 1999

*ARDRA, Amplified rDNA (Ribosomal DNA) Restriction Analysis; RAPD, Random Amplified Polymorphic DNA; AFLP, Amplified Fragment Length Polymorphism; MALDI-TOF MS, Matrix Assisted Laser Desorption Ionization—Time Of Flight Mass Spectrometry*.

## Natural habitat of non-*baumannii Acinetobacter*

### Environment

*Acinetobacter* spp. have for long been described from various environmental sources. In 1994, Wiedman et al. characterized for the first time *A. lwoffii, A. junii*, and *A. johnsonii* in wastewater treatment plants in Germany (Wiedmann-al-Ahmad et al., 1994). Later, Houang et al. investigated soil samples from different areas in Hong Kong and showed that approximately 37% were positive for *Acinetobacter* spp. and that among these bacteria, 27% were *A. pittii* (Houang et al., 2001). Different authors described also new *Acinetobacter* species isolated from activated sludge, sewage treatment plants and raw wastewater in Australia, Portugal, Korea and Pakistan. These species were *Acinetobacter baylyi, Acinetobacter bouvetii, Acinetobacter grimontii, Acinetobacter tjernbergiae, Acinetobacter towneri, Acinetobacter tandoii, Acinetobacter gerneri, Acinetobacter kyonggiensis, Acinetobacter rudis*, and *Acinetobacter pakistanensis* (Carr et al., 2003; Lee and Lee, 2010; Vaz-Moreira et al., 2011; Abbas et al., 2014).

In different studies performed in Korea, authors isolated new *Acinetobacter* species including *Acinetobacter marinus* and *Acinetobacter seohaensis* from seawater (Yoon et al., 2007), *Acinetobacter soli* from forest soil (Kim et al., 2008) as well as *Acinetobacter brisouii* from wetland (Anandham et al., 2010). In another study conducted on soil and artificial environmental samples in Korea, Choi et al. identified *A. calcoaceticus, A. nosocomialis, A. pittii, Acinetobacter* genomic species close to 13TU, *Acinetobacter parvus, Acinetobacter radioresistens, A. soli, A. tandoii, Acinetobacter bereziniae, Acinetobacter schindleri*, and *Acinetobacter* genomic species 15TU, showing a huge diversity of *Acinetobacter* species (Choi et al., 2012). The situation in other countries was slightly different. In Lebanon, Rafei et al. performed studies on several environmental samples to investigate the presence of *Acinetobacter* spp. They showed a prevalence of 18% and discovered that non-*baumannii Acinetobacter*, including *A. pittii* and *A. calcoaceticus* were the most frequently isolated species (Rafei et al., 2015). These findings may highlight the potential role of climatic factors that can affect prevalence of *Acinetobacter* spp. in the environment.

In India, *Acinetobacter indicus* was described for the first time in soil samples collected from hexachlorocyclohexane dump sites (Malhotra et al., 2012). *Acinetobacter kookii* was a novel species isolated from beet fields in Germany, from soil in the Netherlands and in Korea, and from sediments of fish farms in Malaysia and Thailand (Choi et al., 2013). *Acinetobacter venetianus* was a novel species isolated from seawater in Israel, oil in Italy, aquaculture ponds in Denmark and from the sea in Japan (Vaneechoutte et al., 2009). Finally, *Acinetobacter bohemicus* and *Acinetobacter albensis* were two novel species described for the first time in Czech Republic and recovered from natural ecosystems such as soil, mud and water (Krizova et al., 2014, 2015a).

Noteworthy, development of new high throughput sequencing techniques allowed metagenomics studies that could improve our understanding of bacterial microbiota surviving in different environmental sites. For example, *Acinetobacter* spp. were found in soil samples contaminated with petroleum hydrocarbons (Sarma et al., 2004; Bordenave et al., 2007; Obuekwe et al., 2009) and in sediments and water samples in Asian countries collected either from fish pond contaminated with organic waste or from fish and shrimp farms (Huys et al., 2007; Xiong et al., 2015). However, even if metagenomics can provide information on bacterial diversity, in these studies isolates were not characterized at the species level.

In the recent years, several new *Acinetobacter* species have been described in Korea and China. *Acinetobacter antiviralis* and *Acinetobacter oleivorans* were two novel species isolated from tobacco plant roots and rice paddy in Korea (Lee et al., 2009; Kang et al., 2011). In China, *Acinetobacter refrigeratoris, Acinetobacter puyangensis, Acinetobacter qingfengensis, Acinetobacter populi, Acinetobacter guangdongensis*, and *Acinetobacter harbinensis* were six novel species that have been isolated from a refrigerator, popular bark, abandoned lead-zinc ore mine site and surface water of a river respectively (Li et al., 2013, 2014a,b, 2015b; Feng et al., 2014a,b).

Further microbiome studies have been conducted to investigate the bacterial population in the floral nectar of some plants. Interestingly, it has been shown that *Acinetobacter* was the main bacterial taxa founded (Fridman et al., 2012; Álvarez-Pérez and Herrera, 2013). Besides, *Acinetobacter boissieri* and *Acinetobacter nectaris* were two novel species that were isolated from nectar samples of plants in Spain (Álvarez-Pérez et al., 2013).

Recently, it has been shown that the environment could constitute a potential reservoir for *Acinetobacter* spp. resistant isolates. Indeed, carbapenemase and extended-spectrum beta-lactamase producing strains have been isolated from hospital sewage, soil samples around animal farms, but also in polluted rivers (Zong and Zhang, 2013; Maravić et al., 2015; Wang and Sun, 2015; Table 1), highlighting the potential role of these bacteria in the dissemination of antibiotic resistance genes through the environment.

### Food

Presence of *Acinetobacter* spp. in the food chain has also been studied. From 1999, Berlau et al. isolated *A. guillouiae, A. calcoaceticus, A. pittii, A. lwoffii*, and *A. bereziniae* on vegetables purchased from markets in the United Kingdom or harvested from gardens during the summer (Berlau et al., 1999a). In a subsequent study conducted in Hong Kong on vegetables, *A. pittii* and *Acinetobacter genomic species* 10 and 16 have been found (Houang et al., 2001). Different *Acinetobacter* species have also been isolated from fish, meat, cheese and milk samples. In Lebanon, Rafei et al. reported the isolation of non-*baumannii Acinetobacter* including *A. pittii, A. calcoaceticus, A. bereziniae*, and *A. soli* from raw cow meat, raw cheese, raw cow milk and vegetable samples (Rafei et al., 2015), and more recently, they isolated a carbepenem resistant *A. calcoaceticus* from vegetables (Al Atrouni et al., 2016). *Acinetobacter* spp. have been reported in previous studies from milk samples collected from dairy herds In the United States (Jayarao and Wang, 1999) and Kenya (Ndegwa et al., 2001). The isolation rate was 1.3 and 5% respectively. *Acinetobacter* spp. have been reported also from mastitic milk and raw bulk tank milk samples in Korea (Nam et al., 2009; Gurung et al., 2013).

### Animals

While several published studies reported the isolation of *A. baumannii* from animals such as ducks, pigeons, chicken, donkey, rabbits, pets (cats, dogs), mules, livestock (goats, pigs, cattle, caws), horses, lice and arthropods (Gouveia et al., 2008; Hamouda et al., 2008, 2011; Bouvresse et al., 2011; Endimiani et al., 2011; Kempf et al., 2012a,b; Belmonte et al., 2014; Rafei et al., 2015), few studies reported the isolation of non-*baumannii Acinetobacter* from animals. *Acinetobacter* genomic species 15 TU was isolated by Poirel et al. from rectal cow samples in a dairy farm in France (Poirel et al., 2012). More recently, Rafei et al. reported the isolation of *A. pittii, A. calcoaceticus, A. bereziniae, A. johnsonii, A. lwoffii, A. schindleri, A. radioresistens, A. beijerinckii, A. junii, A. gerneri, and Acinetobacter genomic species* 15 TU from animal samples in Lebanon. The strains were isolated mainly from livestocks, horses and pets (Rafei et al., 2015). Smet et al. described for the first time *Acinetobacter gandensis* from horse and cattles (Smet et al., 2014). La Scola et al. reported the detection of *A. anitratus* in lice samples collected from homeless shelters in France (La Scola et al., 2001), and recently *A. radioresistens* and *A. schindleri* were detected from head lice collected from primary school pupils in Thailand (Sunantaraporn et al., 2015). *Acinetobacter* spp. were also detected from aquatic animals (Huys et al., 2007; Geiger et al., 2009) but also in the gut of some arthropods like tsetse fly in Angola, Africa (Guardabassi et al., 1999). Besides, *Acinetobacter apis* was a novel species isolated from the intestinal tract of a honey bee in Korea (Kim et al., 2014).

Furthermore, other studies have been performed to investigate the intestinal ecosystem of fish using metagenomic approaches. As results, *Acinetobacter* was remarquably one of the most abundant genera detected. Indeed, the ability to produce antibacterial compounds against several other species as well as environmental factors and nutrition conditions may affect the bacterial community in the fish intestine and explain the dominance of this group (Hovda et al., 2007; Etyemez and Balcázar, 2015).

Finally, recently, Sun et al. reported the isolation of NDM-1 producing *A. lwoffii* from rectal sample of a cat in China (Sun et al., 2015), suggesting that these companion animals may play a crucial role in the dissemination of multidrug resistant bacteria.

### Human carriage

*Acinetobacter* spp. can be part of the human flora. In a large University Hospital in Cologne, Germany, Seifert et al. performed an epidemiological study to investigate the colonization with *Acinetobacter* spp. of the skin and mucous membranes of hospitalized patients and healthy controls. They showed that the colonization rate was higher in patients than in controls (75 vs. 42.5%) (Seifert et al., 1997). The hands, the groin, toe webs, the forehead and the ears were the most frequently colonized body sites. Almost all the species isolated were non-*baumannii Acinetobacter* including *A. lwoffii* (47%), *A. johnsonii* (21%), *A. radioresistens* (12%), *A. pittii* (11%), and *A. junii* (5%). In contrast, *A. baumannii* and *A. bereziniae* were rarely detected and the authors did not find *A. calcoaceticus* or *A. haemolyticus* on the skin or the mucous membranes (Seifert et al., 1997).

Berlau et al. performed a similar study to investigate the presence of *Acinetobacter* spp. on the skin (forearm, forehead, toe web) of 192 healthy volunteers in the United Kingdom. As in the previous study, they found that the colonization rate was around 40% with *A. lwoffii* being the most frequently isolated species and the forearm being the most frequently colonized area. However, the distribution of the other species was different, *Acinetobacter* genomic species 15BJ (12%), *A. radioresistens* (8%) and only one individual carried the *Acinetobacter baumannii- calcoaceticus* complex (Berlau et al., 1999b). In another study conducted in Hong Kong, Chu et al. showed that the skin carriage rate of student nurses and new nurses from the community was 32 and 66% respectively with *A. pittii* being the most common species (Chu et al., 1999). The authors reported also a potential seasonal variability in skin colonization (Chu et al., 1999). Patil et al. studied skin carriage on six body sites (antecubital fossa, axilla, forehead with hairline, neck, outer surface of nose and toe webs) from volunteers in India. It was found that non-*baumannii Acinetobacter* were the most frequently isolated species including *A. lwoffii, A. junii, A. haemolyticus, A. calcoaceticus*, and *A. pittii*. In this study the antecubital fossa had the highest colonization frequency (48.5%) and the men volunteers were more colonized than the women (Patil and Chopade, 2001).

Likewise, *Acinetobacter* spp. have been also isolated from fecal samples. A study performed by Dijkshoorn et al. in United Kingdom and the Netherlands to investigate the intestinal carriage of *Acinetobacter* spp. showed that from 226 fecal samples collected randomly from the community 38 were positive. The species commonly isolated were: *A. johnsoni*i, *A. guillouiae*, and *A. junii* (Dijkshoorn et al., 2005).

Genomic approaches have also been used to study the bacterial community of some human samples. Thus, Zakharkina et al. reported *Acinetobacter* spp. from airway microbiota of healthy individuals (Zakharkina et al., 2013), while Urbaniak et al. reported the detection of these microorganism from human milk samples (Urbaniak et al., 2014). Recently, in another work conducted to study the microbial diversity of intestinal microbiota of healthy volunteers, Li et al. showed that *Acinetobacter* was present mainly in the duodenum (Li et al., 2015c). According to these findings, we can see the ability of *Acinetobacter* to survive in commensal samples, suggesting that human could constitute a potential reservoir for this opportunistic bacterium. However, the origin and the factors that can influence this colonization remained unclear.

## Global remarks

Referring to these results, we showed here that the environment is the main reservoir of *Acinetobacter* spp. and interestingly the bacteria have been mainly isolated from sites in contact with human, animal or in areas polluted with hydrocarbon. Therefore, it has been suggested that *Acinetobacter* spp. belong to the small minority of species that are able to dominate within an open habitat (Cray et al., 2013). Indeed, the microorganisms are exposed in the environment to multiple factors that affect their growth and act as stress parameters such as desiccation conditions, temperature, air humidity and other parameters that are subjected to dynamic changes. Unlike some other Gram negative bacteria, *Acinetobacter* spp. are able to survive in a dry environment for long periods of time and support desiccation conditions (Wendt et al., 1997; Wagenvoort and Joosten, 2002). This tolerance may be due to different mechanisms such as over expression of proteins involved in the antimicrobial resistance, efflux pumps, down regulation of proteins involved in the cell cycle, transcription and translation in order to enter in a dormant state (Gayoso et al., 2014). Furthermore, hydrocarbons and polysaccharides are macromolecules available in the environment and may constitute a primary substrate for these microorganisms. *Acinetobacter* species can catabolize the polysaccharides *via* the production of xylanase which is a key enzyme to degrade complex extracellular substances such as hemicelluloses. It has also been shown that pollution of environmental sites either with fuel oil or metals can affect the microbial diversity and only few types of bacteria such as *Acinetobacter* spp. were able to resist and dominate such polluted areas (Bordenave et al., 2007; Zhao et al., 2014). Moreover, these bacteria are able to degrade various pollutants and organic compounds and have an important role in environmental bioremediation (Adegoke et al., 2012; Cray et al., 2013). Finally, *Acinetobacter* spp. have developed strategies to inhibit the growth of competing species either by acidification of the environment (secretion of organic acids) or by production inhibitory biosurfactants (Cray et al., 2013).

In this review, we showed also that the use of DNA based methods contribute to the progress in the field of the diversity of the genus *Acinetobacter*. As a result, a large number of well characterized species were available and *Acinetobacter* remains an interesting model for taxonomist to study the natural diversity as well as the evolutionary history of this bacterium. In fact, recent studies suggested that climatic changes and pollution have the potential to alter the species distribution in the environment (Coelho et al., 2013). Other theories consider that evolution of species may be the direct response to climatic modifications (Hoffmann and Sgrò, 2011). These findings raise many questions whether description of new *Acinetobacter* species was the result of those ecological changes. On the other hand, there is an important question that remains unclearly answered: could these newly described *Acinetobacter* species have a potential role in human infection? In fact, several studies showed that uncommon and newly described *Acinetobacter* species such as *A. septicus* and *A. bereziniae* were involved in human infection and some of them were resistant to carbapenems (Kilic et al., 2008; Kuo et al., 2010; Sung et al., 2014). Moreover, other studies conducted in France, Croatia, Japan and China reported the detection of multidrug resistant strains of *A. schindleri, A. guillouiae, A. soli, A. ursingii* and *A. beijerinckii* isolated from clinical samples (Dortet et al., 2006; Bošnjak et al., 2014; Endo et al., 2014; Fu et al., 2015; Quiñones et al., 2015). Based on these results, one can presume that other species of *Acinetobacter* will be discovered soon in human infections thanks to more efficient molecular techniques used for bacterial identification.

In conclusion, even if the present data derived from only few studies, it seems that almost all of the *Acinetobacter* species are widely distributed in nature and that the contaminated environment may enhance the growth of these microorganisms. Further studies are nevertheless required to understand the behavior of *Acinetobacter* spp. and to elucidate the mode of transmission of those bacteria from these different habitats to humans.

## Author contributions

AA, MJ, MH, and MK contributed to the conception and design of the work, and to the acquisition and interpretation of the data. All authors contributed to the drafting of the manuscript and approved the final version to be published.

### Conflict of interest statement

The authors declare that the research was conducted in the absence of any commercial or financial relationships that could be construed as a potential conflict of interest.
